# Supplementary material for: Human oligodendrocyte progenitor cells mediate synapse elimination through TAM receptor activation
Source: Nat Commun. 2025 Dec 5;16:10612. doi: 10.1038/s41467-025-66521-1 (PMC12680777; doi:10.1038/s41467-025-66521-1)
Supplement: Supplementary file 1 — Supplementary Information [file 41467_2025_66521_MOESM1_ESM.pdf]

Human oligodendrocyte progenitor cells mediate synapse elimination through TAM receptor activation

Asimenia Gkogka[1], Susmita Malwade[1], Marja Koskuvi[1], Sohvi Ohtonen[1], Ellinor Molnar[1], Raj Bose[2], Sandra Ceccatelli[2], Jari Koistinaho[3], Jari Tiihonen[3,4,5], Martin Schalling[6], Samudyata Samudyata[‡1], Carl M. Sellgren[*‡1,5]

[1]Department of Physiology and Pharmacology, Karolinska Institutet, Stockholm, Sweden.

[2]Department of Neuroscience, Karolinska Institutet, Stockholm, Sweden.

[3]Neuroscience Center, HiLIFE, and Drug Research Program, Division of Pharmacology and Pharmacotherapy, University of Helsinki, Helsinki.

[4]Department of Forensic Psychiatry, University of Eastern Finland, Niuvanniemi Hospital, Kuopio, Finland.

[5]Center for Psychiatry Research, Department of Clinical Neuroscience, Karolinska Institutet and Stockholm Health Care Services, Stockholm County Council, Stockholm, Sweden.

[6]Department of Molecular Medicine and Surgery, Karolinska Institutet and Center for Molecular Medicine, Karolinska University Hospital, Stockholm, Sweden.

‡These authors jointly supervised this work.

*Correspondence: carl.sellgren@ki.se (C.M.S).

SUPPLEMENTARY INFORMATION

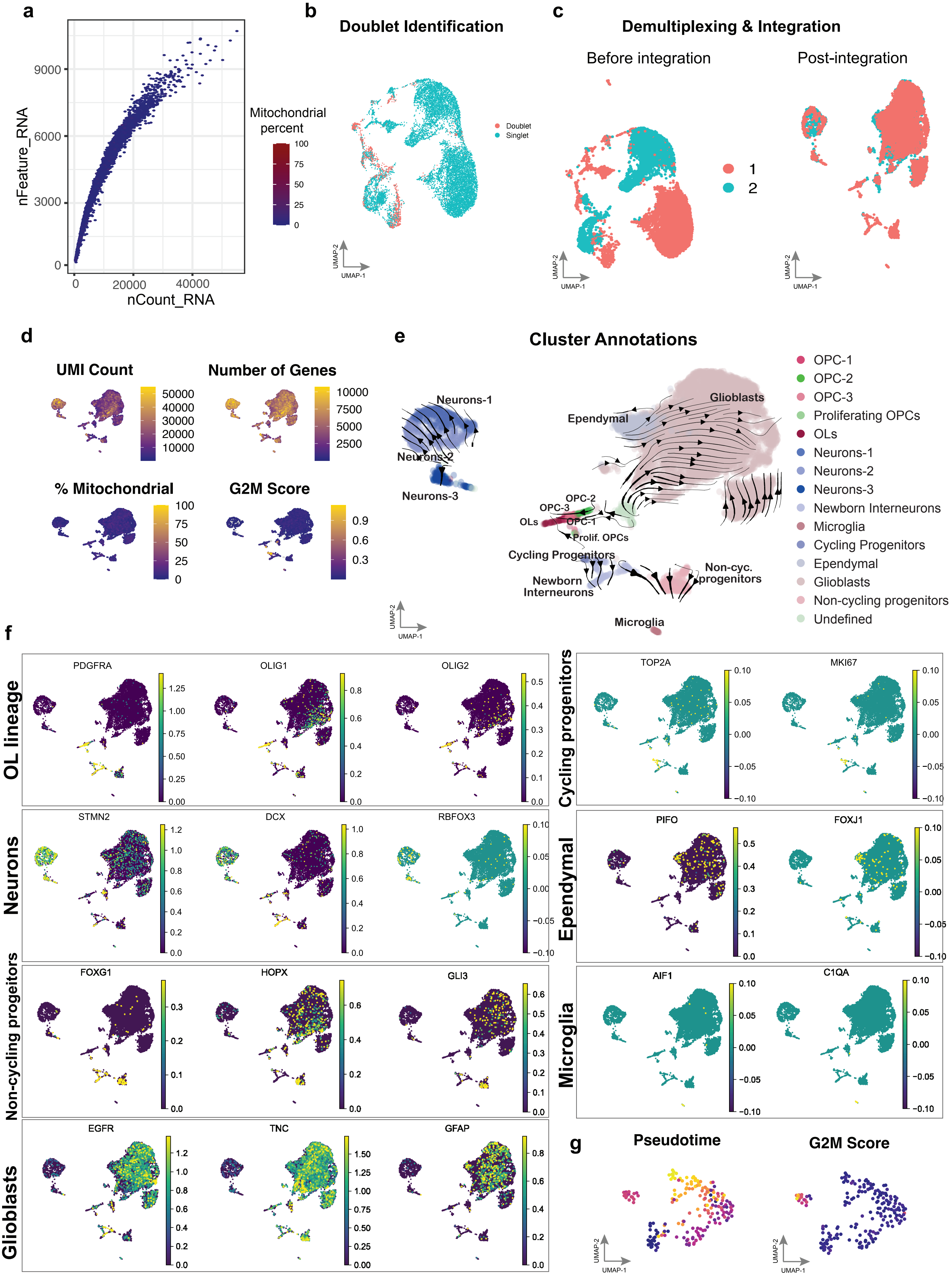
a
Mitochondrial percent
nFeature_RNA
nCount_RNA
b
Doublet Identification
Doublet
Singlet
c
Demultiplexing & Integration
Before integration
Post-integration
d
UMI Count
Number of Genes
% Mitochondrial
G2M Score
e
Cluster Annotations
Neurons-1
Neurons-2
Neurons-3
Ependymal
Glioblasts
OPC-2
OPC-3
OPC-1
OLs
Prolif. OPCs
Cycling Progenitors
Newborn Interneurons
Non-cyc. progenitors
Microglia
OPC-1
OPC-2
OPC-3
Proliferating OPCs
OLs
Neurons-1
Neurons-2
Neurons-3
Newborn Interneurons
Microglia
Cycling Progenitors
Ependymal
Glioblasts
Non-cycling progenitors
Undefined
f
OL lineage
PDGFRA
OLIG1
OLIG2
Neurons
STMN2
DCX
RBFOX3
Non-cycling progeitors
FOXG1
HOPX
GLI3
Glioblasts
EGFR
TNC
GFAP
Cycling progenitors
TOP2A
MKI67
Ependymal
PIFO
FOXJ1
Microglia
AIF1
C1QA
g
Pseudotime
G2M Score
UMAP-1
UMAP-2

Supplementary Fig. 1: snRNA-seq quality control metrics and clustering of cells isolated from multi-lineage forebrain organoids at DIV 250. **a** Scatter plot denoting number of transcripts captured (x-axis) versus the number of genes captured (y-axis) per cell within the dataset after quality control. Data points represent individual cells colored by the percentage of mitochondrial transcripts. **b** UMAP plot showing each cell colored by the doublet status assigned by DoubletFinder. Assigned doublets were filtered out for downstream applications. **c** UMAP embedding of the dataset with each cell colored by subject line, demultiplexed by genotype (see methods), as a confounder (left). Cells from individual cell lines were integrated (see methods) to obtain the final embedding for downstream analysis (right). **d** UMAP plots of the final embedding showing quality control metrics such as number of transcripts (UMI count), number of genes, percentage of mitochondrial transcripts, and G2M phase score for individual cells, showing uniform distribution and no confounding effects. **e** UMAP plot visualizing the distribution of clusters colored by the cell type annotations used for downstream analyses. Black lines and arrows denoted RNA velocity streamlines. **f** UMAP plots showing the distribution of canonical marker gene expression for broad cell types during brain development across individual cells. Color scale represents the average scaled expression for single genes. **g** UMAP plot of the cells obtained from OL-lineage (in Figure 1h, i) colored by computed pseudotime (left) and the cell cycle score for G2M phase (right).

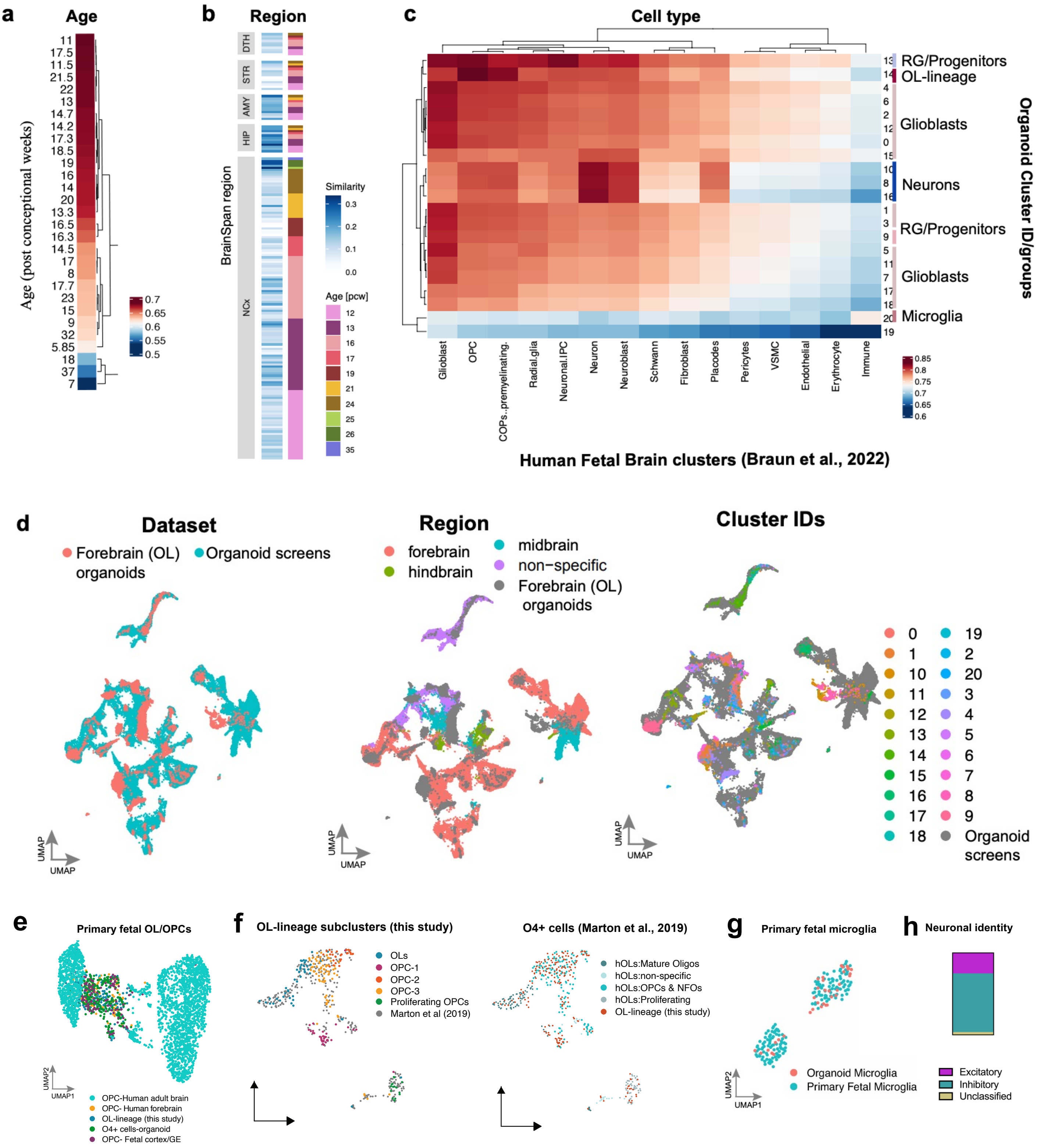

a
Age
Age (post conceptional weeks)
b
Region
BrainSpan region
DTH
STR
AMY
HIP
NCx
Similarity
Age [pcw]
c
Cell type
Glioblast
OPC
COPs..premyelinating.
Radial.glia
Neuronal.IPC
Neuron
Neuroblast
Schwann
Fibroblast
Placodes
Pericytes
VSMC
Endothelial
Erythrocyte
Immune
RG/Progenitors
OL-lineage
Glioblasts
Neurons
RG/Progenitors
Glioblasts
Microglia
Organoid Cluster ID/groups
Human Fetal Brain clusters (Braun et al., 2022)
d
Dataset
Forebrain (OL) organoids
Organoid screens
Region
forebrain
hindbrain
midbrain
non−specific
Forebrain (OL) organoids
Cluster IDs
Organoid screens
UMAP
e
Primary fetal OL/OPCs
OPC-Human adult brain
OPC- Human forebrain
OL-lineage (this study)
O4+ cells-organoid
OPC- Fetal cortex/GE
f
OL-lineage subclusters (this study)
OLs
OPC-1
OPC-2
OPC-3
Proliferating OPCs
Marton et al (2019)
O4+ cells (Marton et al., 2019)
hOLs:Mature Oligos
hOLs:non-specific
hOLs:OPCs & NFOs
hOLs:Proliferating
OL-lineage (this study)
g
Primary fetal microglia
Organoid Microglia
Primary Fetal Microglia
h
Neuronal identity
Excitatory
Inhibitory
Unclassified

Supplementary Fig. 2: Reference mapping and integration of forebrain organoid data with human brain datasets. Reference mapping utilizing Spearman-ranked correlation of forebrain organoids to reference transcriptomes of **a** primary fetal cortex and GE (Nowakowski *et al*[1]) for fetal age, **b** spatial similarity to human micro dissected brain from BrainSpan (https://www.brainspan.org/), and **c** large-scale dataset of human fetal brain (Braun *et al*[2]) for cell-type similarities. **d** UMAP embedding of the integrated space of forebrain organoid dataset (this study) with region-directed brain organoids dataset (Amin *et al*[3]), including forebrain, midbrain and hindbrain organoids. UMAP plots show cells colored by dataset (left), regional specification (middle), and cluster IDs from Fig. 1g (right). **e** UMAP plot of the integrated space of single-cell datasets of OPCs from human adult brain (Hodge et al[4]), human forebrain (van Bruggen *et al*[5]), human developing cortex (Nowakowski *et al*[1]), organoid-isolated O4+ cells (Marton *et al*[6]), and OL-lineage cells (cluster 14) from forebrain organoids from this study. **f** UMAP plot of the integrated space of OL-lineage cells (cluster 14) from forebrain organoids (this study) with a reference dataset of organoid-isolated O4+ cells (Marton *et al*[6]). Cells are colored by the annotated OL subclusters from Figure 1i of this study (left) and by the original annotations of the reference dataset (right). **g** UMAP plot of the integrated space of microglia (cluster 20) from forebrain organoids with human fetal microglia (Nowakowski *et al*[1]) showing an overlap across two microglia states from the fetal brain. **h** Quantification of excitatory and inhibitory neuronal composition based on curated gene signatures derived from human fetal cortical development (Velmeshev *et al*[7]), which classified 71.2% of neurons as inhibitory and 24.8% as excitatory, and the remaining 4.0% unclassified.

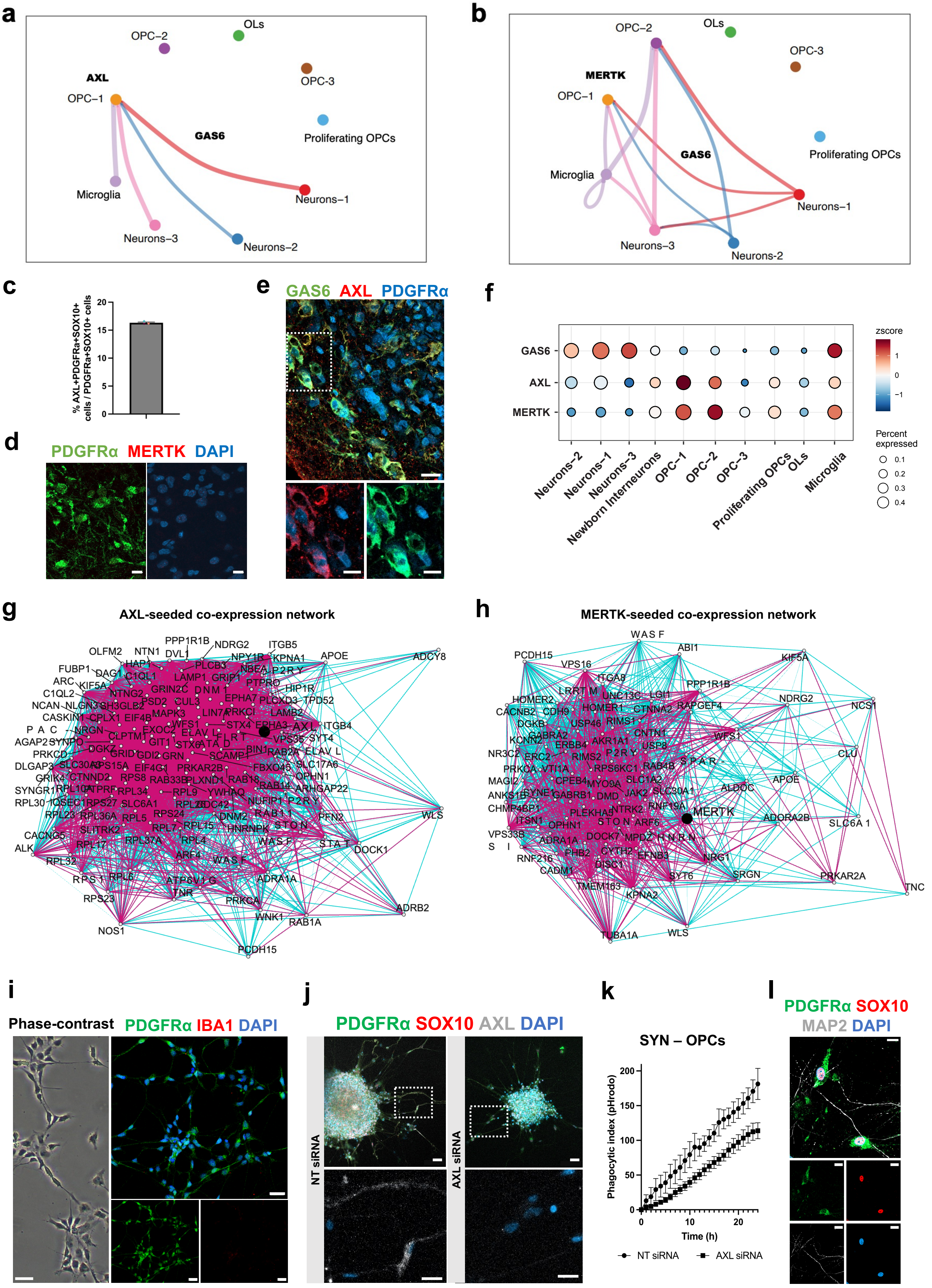
a
OPC–2
OLs
OPC–3
AXL
OPC–1
GAS6
Proliferating OPCs
Microglia
Neurons–1
Neurons–3
Neurons–2
b
OPC–2
OLs
OPC–3
MERTK
OPC–1
GAS6
Proliferating OPCs
Microglia
Neurons–1
Neurons–3
Neurons-2
c
% AXL+PDGFRα+SOX10+ cells / PDGFRα+SOX10+ cells
20
15
10
5
0
d
PDGFRα MERTK DAPI
e
GAS6 AXL PDGFRα
f
GAS6
AXL
MERTK
Neurons–2
Neurons–1
Neurons–3
Newborn Interneurons
OPC–1
OPC–2
OPC–3
Proliferating OPCs
OLs
Microglia
zscore
1
0
−1
Percent expressed
0.1
0.2
0.3
0.4
g
AXL-seeded co-expression network
h
MERTK-seeded co-expression network
i
Phase-contrast
PDGFRα IBA1 DAPI
j
PDGFRα SOX10 AXL DAPI
NT siRNA
AXL siRNA
k
SYN – OPCs
Phagocytic index (pHrodo)
200
150
100
50
0
0
10
20
Time (h)
NT siRNA
AXL siRNA
l
PDGFRα SOX10
MAP2 DAPI

Supplementary Fig. 3: **Expression and co-expression networks of TAM receptors, and functional analysis of hiPSC-derived OPCs using synaptosome assay**. **a** GAS6 secreted by neurons and microglia predicted to interact with AXL receptors on OPC-1 population in the organoids, based on ligand-receptor expression analysis using CellChat. **b** GAS6 secreted by neurons and microglia predicted to interact with MERTK receptors on OPC-1 and OPC-2 populations in the organoids, based on ligand-receptor expression analysis using CellChat. **c** Quantification of AXL-expressing cells out of double-positive PDGFRα+SOX10+ cells in the forebrain organoid model. **d** Representative IHC image showing absence of MERTK in OPCs (PDGFRα) within the human forebrain organoid at DIV 130. **e** Representative IHC image revealing presence of GAS6+ signal on AXL-expressing OPCs. **f** Expression of TAM RTKs AXL and MERTK as well as their ligand GAS6 among neurons, microglia and OL-lineage cells in forebrain organoids. **g-h** Unsupervised co-expression networks of AXL (**g**) or MERTK (**h**) used as the "seed gene" (black dot) constructed using RNA-seq data from primary human brain tissue (GTEx Portal[8]). Red lines represent positive network; blue lines represent negative network of the respective seed gene and edges denote gene-gene co-expression. Transcriptome-wide significance was calculated at FDR-adjusted *P*-values<0.05. Pearson's correlation coefficient threshold was set to 0.3 and the network was restricted to synapse-related genes from the SynGO Database[9] for visualization purposes. **i** Brightfield image of hiPSC-derived OPCs displaying typical bipolar morphology (left) and representative IHC image of cells expressing the OPC marker PDGFRα but not the microglial marker IBA1 (right). **j** Representative IHC images of OPC colonies (PDGFRα, SOX10) upon 4-day treatment with control (NT) siRNA or siRNA targeted against the *Axl* mRNA, showing decreased expression of AXL protein. **k** Quantification of real-time, live, fluorescent imaging of synaptosome engulfment assay of OPCs exposed to pHrodo-labelled synaptosomes upon treatment with non-targeting RNA (NT siRNA, control) or RNA targeting AXL mRNA (AXL siRNA,

knockdown) over a 24-hour period. **l** Representative immunostaining of neuron-OPC co-cultures at day 26 of differentiation, with PDGFRα+SOX10+ cells and MAP2+ neurites. Scale bars: 10 μm (**d**, **e**), 5 μm (magnification of **e**), 20 μm (**i**, **l**), 30 μm (**j**).

SUPPLEMENTARY REFERENCES

1. Nowakowski, T. J. *et al.* Spatiotemporal gene expression trajectories reveal developmental hierarchies of the human cortex. *Science (1979)* **358**, 1318–1323 (2017).
2. Braun, E. *et al.* Comprehensive cell atlas of the first-trimester developing human brain. *bioRxiv* 2022.10.24.513487-2022.10.24.513487 (2022) doi:10.1101/2022.10.24.513487.
3. Amin, N. D. *et al.* Generating human neural diversity with a multiplexed morphogen screen in organoids. *bioRxiv* 2023.05.31.541819-2023.05.31.541819 (2023) doi:10.1101/2023.05.31.541819.
4. Hodge, R. D. *et al.* Conserved cell types with divergent features in human versus mouse cortex. *Nature 2019 573:7772* **573**, 61–68 (2019).
5. van Bruggen, D. *et al.* Developmental landscape of human forebrain at a single-cell level identifies early waves of oligodendrogenesis. *Dev Cell* **57**, 1421-1436.e5 (2022).
6. Marton, R. M. *et al.* Differentiation and maturation of oligodendrocytes in human three-dimensional neural cultures. *Nat Neurosci* **22**, 484–491 (2019).
7. Velmeshev, D. *et al.* Single-cell analysis of prenatal and postnatal human cortical development. *Science (1979)* **382**, (2023).
8. Lonsdale, J. *et al.* The Genotype-Tissue Expression (GTEx) project. *Nature Genetics* vol. 45 580–585 Preprint at https://doi.org/10.1038/ng.2653 (2013).
9. Koopmans, F. *et al.* SynGO: An Evidence-Based, Expert-Curated Knowledge Base for the Synapse. *Neuron* **103**, 217-234.e4 (2019).
