# Supplementary material for: Human oligodendrocyte progenitor cells mediate synapse elimination through TAM receptor activation
Source: Nat Commun. 2025 Dec 5;16:10612. doi: 10.1038/s41467-025-66521-1 (PMC12680777; doi:10.1038/s41467-025-66521-1)
Supplement: Supplementary file 2 — Description of Additional Supplementary Files [file 41467_2025_66521_MOESM2_ESM.pdf]

DESCRIPTION OF ADDITIONAL SUPPLEMENTARY FILES

Supplementary Data 1. Cluster identification and characterization in forebrain organoids based on reference mapping and gene expression analysis.

Supplementary Data 2. Co-expression networks of TAM receptor AXL in primary human brain tissue.

Supplementary Data 3. Co-expression networks of TAM receptor MERTK in primary human brain tissue.

Supplementary Data 4. List of reagents and resources used in this study.
