# Supplementary material for: Human oligodendrocyte progenitor cells mediate synapse elimination through TAM receptor activation
Source: Nat Commun. 2025 Dec 5;16:10612. doi: 10.1038/s41467-025-66521-1 (PMC12680777; doi:10.1038/s41467-025-66521-1)
Supplement: Supplementary file 7 — Reporting Summary [file 41467_2025_66521_MOESM7_ESM.pdf]

nature portfolio

Corresponding author(s): Carl M. Sellgren
Last updated by author(s): Nov 6, 2025

Reporting Summary

Nature Portfolio wishes to improve the reproducibility of the work that we publish. This form provides structure for consistency and transparency in reporting. For further information on Nature Portfolio policies, see our Editorial Policies and the Editorial Policy Checklist.

Statistics

For all statistical analyses, confirm that the following items are present in the figure legend, table legend, main text, or Methods section.

n/a	Confirmed	
☐	☒	The exact sample size (*n*) for each experimental group/condition, given as a discrete number and unit of measurement
☐	☒	A statement on whether measurements were taken from distinct samples or whether the same sample was measured repeatedly
☐	☒	The statistical test(s) used AND whether they are one- or two-sided<br>*Only common tests should be described solely by name; describe more complex techniques in the Methods section.*
☒	☐	A description of all covariates tested
☐	☒	A description of any assumptions or corrections, such as tests of normality and adjustment for multiple comparisons
☐	☒	A full description of the statistical parameters including central tendency (e.g. means) or other basic estimates (e.g. regression coefficient) AND variation (e.g. standard deviation) or associated estimates of uncertainty (e.g. confidence intervals)
☐	☒	For null hypothesis testing, the test statistic (e.g. *F*, *t*, *r*) with confidence intervals, effect sizes, degrees of freedom and *P* value noted<br>*Give P values as exact values whenever suitable.*
☒	☐	For Bayesian analysis, information on the choice of priors and Markov chain Monte Carlo settings
☒	☐	For hierarchical and complex designs, identification of the appropriate level for tests and full reporting of outcomes
☐	☒	Estimates of effect sizes (e.g. Cohen's *d*, Pearson's *r*), indicating how they were calculated

Our web collection on statistics for biologists contains articles on many of the points above.

Software and code

Policy information about availability of computer code

Data collection	No software was used for data collection
Data analysis	All custom code used for the single-nucleus RNA sequencing analysis and data visualisation is available at Zenodo under DOI: 10.5281/zenodo.17418028 and in the GitHub repository at https://github.com/SellgrenLab/organoid-oligodendrocyte.<br>List of softwares used: GraphPad Prism (v8.0.0), Zeiss Zen (Blue edition) (v2.3), ImageJ Fiji (v2.0.0), Imaris (v10.0.0), Microsoft Excel, Adobe Illustrator, QuantStudio Real Time PCR software (v1.3), IncuCyte ZOOM Live Imaging (v2016A), AxIS Software Spontaneous Neural Configuration, Cellranger (v6.1.2), Seurat (v4.0), SouporCell, DoubletFinder (v3), Velocyto (v0.17.15), ScVelo (v0.2.5), CellRank (v1), Harmony (v0.1.1), CellChat (v1.6.0), clustifyr, R (v4.2.2), Python (v3.7.12).

For manuscripts utilizing custom algorithms or software that are central to the research but not yet described in published literature, software must be made available to editors and reviewers. We strongly encourage code deposition in a community repository (e.g. GitHub). See the Nature Portfolio guidelines for submitting code & software for further information.

Data

Policy information about availability of data

All manuscripts must include a data availability statement. This statement should provide the following information, where applicable:

- Accession codes, unique identifiers, or web links for publicly available datasets
- A description of any restrictions on data availability
- For clinical datasets or third party data, please ensure that the statement adheres to our policy

Single-nucleus RNA sequencing analysis and visualisation were performed in R (v4.2.2) and Python (v3.7.12) on macOS (Ventura 13.4). The sequencing data generated in this study have been deposited in the GEO database under the accession number GSE242275. Previously published datasets used for reference mapping and comparative analyses were obtained from Braun et al. (2022) [https://ega-archive.org/datasets/EGAD00001006049, accession number EGAS00001004107], Cameron et al. (2022) [https://figshare.com/articles/dataset/11629311, accession number EGAS00001006537], Nowakowski et al. (2017) [https://www.ncbi.nlm.nih.gov/projects/gap/cgi-bin/study.cgi?study_id=phs000989.v6.p1, accession number phs000989.v3], Marton et al. (2019) [https://www.ncbi.nlm.nih.gov/geo/query/acc.cgi?acc=GSE115011, accession number GSE115011], and Amin et al. (2024), [https://www.ncbi.nlm.nih.gov/geo/query/acc.cgi?acc=GSE233574, accession number GSE233574]. All microscopy- and qPCR-based quantifications, for which GraphPad Prism (v8.0) was used, are provided within the paper and its Source Data file.

Research involving human participants, their data, or biological material

Policy information about studies with human participants or human data. See also policy information about sex, gender (identity/presentation), and sexual orientation and race, ethnicity and racism.

Reporting on sex and gender	*Use the terms sex (biological attribute) and gender (shaped by social and cultural circumstances) carefully in order to avoid confusing both terms. Indicate if findings apply to only one sex or gender; describe whether sex and gender were considered in study design; whether sex and/or gender was determined based on self-reporting or assigned and methods used. Provide in the source data disaggregated sex and gender data, where this information has been collected, and if consent has been obtained for sharing of individual-level data; provide overall numbers in this Reporting Summary. Please state if this information has not been collected. Report sex- and gender-based analyses where performed, justify reasons for lack of sex- and gender-based analysis.*
Reporting on race, ethnicity, or other socially relevant groupings	*Please specify the socially constructed or socially relevant categorization variable(s) used in your manuscript and explain why they were used. Please note that such variables should not be used as proxies for other socially constructed/relevant variables (for example, race or ethnicity should not be used as a proxy for socioeconomic status). Provide clear definitions of the relevant terms used, how they were provided (by the participants/respondents, the researchers, or third parties), and the method(s) used to classify people into the different categories (e.g. self-report, census or administrative data, social media data, etc.) Please provide details about how you controlled for confounding variables in your analyses.*
Population characteristics	*Describe the covariate-relevant population characteristics of the human research participants (e.g. age, genotypic information, past and current diagnosis and treatment categories). If you filled out the behavioural & social sciences study design questions and have nothing to add here, write "See above."*
Recruitment	*Describe how participants were recruited. Outline any potential self-selection bias or other biases that may be present and how these are likely to impact results.*
Ethics oversight	*Identify the organization(s) that approved the study protocol.*

Note that full information on the approval of the study protocol must also be provided in the manuscript.

Field-specific reporting

Please select the one below that is the best fit for your research. If you are not sure, read the appropriate sections before making your selection.

[x] Life sciences [ ] Behavioural & social sciences [ ] Ecological, evolutionary & environmental sciences

For a reference copy of the document with all sections, see nature.com/documents/nr-reporting-summary-flat.pdf

Life sciences study design

All studies must disclose on these points even when the disclosure is negative.

Sample size	No statistical methods were used to pre-determine sample sizes. Our sample sizes were estimated empirically, based on those reported in previous publications in the field.
Data exclusions	No data points were excluded. For single nucleus analysis, we performed strict quality filtering and excluded individual cells that did not contain high quality data. For the details and filtering criteria see: Methods - snRNA-seq data processing and analyses.
Replication	Experiments were performed using 5 individual iPSC lines derived from 5 healthy subjects. For each type of experiment, multiple organoids (at least n=4) were collected from each differentiation for each time-point per subject. Data were collected from 2 individual differentiations of

	each iPSC line for the organoid experiments, and from 2 individual differentiations of each iPSC line for the 2D OPC experiments. The number of replicates for each type of experiment are indicated in the respective sections of the manuscript.
Randomization	iPSC lines and organoids were randomly selected for each type of experiment.
Blinding	Organoids were picked blindly for each time-point, while the investigators were not blinded to the age of the organoids. The investigators were blinded to the treatment condition for the 2D OPC experiments using the inhibitor as well as the siRNA.

Reporting for specific materials, systems and methods

We require information from authors about some types of materials, experimental systems and methods used in many studies. Here, indicate whether each material, system or method listed is relevant to your study. If you are not sure if a list item applies to your research, read the appropriate section before selecting a response.

Materials & experimental systems

n/a	Involved in the study	
☐	☒	Antibodies
☐	☒	Eukaryotic cell lines
☒	☐	Palaeontology and archaeology
☒	☐	Animals and other organisms
☒	☐	Clinical data
☒	☐	Dual use research of concern
☒	☐	Plants

Methods

n/a	Involved in the study	
☒	☐	ChIP-seq
☒	☐	Flow cytometry
☒	☐	MRI-based neuroimaging

Antibodies

Antibodies used

A detailed list of all antibodies used in the study is provided in Supplementary Table 5.

Primary antibodies are as follow:
Goat anti-PDGFRα (1:100), R & D Systems, Cat# AF-307-NA
Mouse anti-PDGFRα (1:50), R & D Systems, Cat# 221-AA-025
Goat anti-OLIG2 (1:100), R & D Systems, Cat# AF2418
Rabbit anti-NG2 (1:100), Cell Signaling Technology, Cat# 43916
Mouse anti-MBP (1:200), R & D Systems, Cat# MAB42282
Rabbit anti-NKX2-1 (1:300), Abcam, Cat# ab76013
Goat anti-IBA1 (1:100), Novus Biologicals, Cat# NB100-1028
Rabbit anti-IBA1 (1:300) , Wako, Cat# 019-19741
Rabbit anti-GFAP (1:500), Agilent Dako, Cat# Z0334
Mouse anti-AQP4 (1:100), Santa Cruz Biotechnology, Cat# sc-32739
Chicken anti-MAP2 (1:500), Abcam, Cat# ab5392
Rabbit anti-vGLUT1 (1:100), Abcam, Cat# ab272913
Rabbit anti-GABA (1:500), Sigma, Cat# A2052
Rabbit anti-SYN1 (1:200), Synaptic Systems, Cat# 106 103
Mouse anti-PSD-95 (1:100), Abcam, Cat# Ab13552
Mouse anti-GEPHYRIN (1:100), Synaptic Systems, Cat# 147021
Rabbit anti-LAMP2 (1:200), Thermo Fisher Scientific, Cat# PA1-655
Rabbit anti-GAS6 (1:500), Bioss, Cat# BS-7549R
Rabbit anti-MERTK (1:100), Abcam, Cat# ab52968
Goat anti-AXL (1:200), R & D Systems, Cat# AF154
Primary antibodies are as follow:
Goat anti-PDGFRα (1:100), R & D Systems, Cat# AF-307-NA
Mouse anti-PDGFRα (1:50), R & D Systems, Cat# 221-AA-025
Goat anti-OLIG2 (1:100), R & D Systems, Cat# AF2418
Rabbit anti-NG2 (1:100), Cell Signaling Technology, Cat# 43916
Mouse anti-MBP (1:200), R & D Systems, Cat# MAB42282
Rabbit anti-NKX2-1 (1:300), Abcam, Cat# ab76013
Goat anti-IBA1 (1:100), Novus Biologicals, Cat# NB100-1028
Rabbit anti-IBA1 (1:300) , Wako, Cat# 019-19741
Rabbit anti-GFAP (1:500), Agilent Dako, Cat# Z0334
Mouse anti-AQP4 (1:100), Santa Cruz Biotechnology, Cat# sc-32739
Chicken anti-MAP2 (1:500), Abcam, Cat# ab5392
Rabbit anti-vGLUT1 (1:100), Abcam, Cat# ab272913
Rabbit anti-GABA (1:500), Sigma, Cat# A2052
Rabbit anti-SYN1 (1:200), Synaptic Systems, Cat# 106 103
Mouse anti-PSD-95 (1:100), Abcam, Cat# Ab13552

Mouse anti-GEPHYRIN (1:100), Synaptic Systems, Cat# 147021
Rabbit anti-LAMP2 (1:200), Thermo Fisher Scientific, Cat# PA1-655
Rabbit anti-GAS6 (1:500), Bioss, Cat# BS-7549R
Rabbit anti-MERTK (1:100), Abcam, Cat# ab52968
Goat anti-AXL (1:200), R & D Systems, Cat# AF154
Mouse anti-TYRO3 (1:100), R & D Systems, Cat# MAB859.

Secondary antibodies are as follow:
Donkey anti-mouse Alexa Fluor 488 (1:500), Thermo Fisher Scientific, Cat# R37114
Donkey anti-rabbit Alexa Fluor 555 (1:500), Thermo Fisher Scientific, Cat# A31572
Donkey anti-goat Alexa Fluor 555 (1:500), Thermo Fisher Scientific, Cat# A21447
Donkey anti-rat Alexa Fluor 555 (1:500), Thermo Fisher Scientific , Cat# SAA-10029
Goat anti-chicken Alexa Fluor 555 (1:500), Thermo Fisher Scientific, Cat# A21449
Goat anti-mouse Alexa Fluor 488 (1:500), Thermo Fisher Scientific, Cat# A28175
Goat anti-rabbit Alexa Fluor 555 (1:500), Thermo Fisher Scientific, Cat# A21428

Validation

All antibodies are commercially available and validated, with several of them having been used by us and others on human brain organoids.
According to the manufacturer's website:
The goat anti-PDGFRα antibody has been cited in 56 publications.
The mouse anti-PDGFRα antibody has been cited in 7 publications.
The goat anti-OLIG2 antibody has been cited in 193 publications.
The rabbit anti-NG2 antibody has been cited in 4 publications.
The mouse anti-MBP antibody has been cited in 17 publications.
The rabbit anti-NKX2-1 antibody has been cited in 175 publications.
The goat anti-IBA1 antibody has been cited in 424 publications.
The rabbit anti-GFAP antibody has been cited in 3904 publications.
The mouse anti-AQP4 antibody has been cited in 41 publications.
The chicken anti-MAP2 antibody has been cited in 787 publications.
The rabbit anti-vGLUT1 antibody has been cited in 42 publications.
The rabbit anti-GABA antibody has been cited in 879 publications.
The rabbit anti-SYN1 antibody has been cited in 44 publications.
The mouse anti-PSD-95 antibody has been cited in 54 publications.
The mouse anti-GEPHYRIN antibody has been cited in 109 publications.
The rabbit anti-LAMP2 antibody has been cited in 42 publications.
The rabbit anti-GAS6 antibody has been cited in 5 publications.
The rabbit anti-MERTK antibody has been cited in 61 publications.
The goat anti-AXL antibody has been cited in 118 publications.
The mouse anti-TYRO3 antibody has been cited in 11 publications.

Eukaryotic cell lines

Policy information about cell lines and Sex and Gender in Research

Cell line source(s)

Dermal biopsies were collected from 3 male and 2 female healthy individuals.

Authentication

Describe the authentication procedures for each cell line used OR declare that none of the cell lines used were authenticated.

Mycoplasma contamination

All cell lines were assessed for Mycoplasma contamination and tested negative.

Commonly misidentified lines
(See ICLAC register)

No commonly misidentified cell lines were used.

Plants

Seed stocks

Report on the source of all seed stocks or other plant material used. If applicable, state the seed stock centre and catalogue number. If plant specimens were collected from the field, describe the collection location, date and sampling procedures.

Novel plant genotypes

Describe the methods by which all novel plant genotypes were produced. This includes those generated by transgenic approaches, gene editing, chemical/radiation-based mutagenesis and hybridization. For transgenic lines, describe the transformation method, the number of independent lines analyzed and the generation upon which experiments were performed. For gene-edited lines, describe the editor used, the endogenous sequence targeted for editing, the targeting guide RNA sequence (if applicable) and how the editor was applied.

Authentication

Describe any authentication procedures for each seed stock used or novel genotype generated. Describe any experiments used to assess the effect of a mutation and, where applicable, how potential secondary effects (e.g. second site T-DNA insertions, mosiacism, off-target gene editing) were examined.
